# Research on the Influence of the Manufacturing Process Conditions of Iron Sintered with the Addition of Layered Lubricating Materials on its Selected Properties

**DOI:** 10.3390/ma13214782

**Published:** 2020-10-26

**Authors:** Wieslaw Urbaniak, Tomasz Majewski, Ryszard Wozniak, Judyta Sienkiewicz, Jozef Kubik, Aneta D. Petelska

**Affiliations:** 1 Faculty of Mechatronics, Kazimierz Wielki University, Chodkiewicz 30, 85-867 Bydgoszcz, Poland; kubik@ukw.edu.pl; 2 Faculty of Mechatronics, Armament and Aerospace of MUT, Military University of Technology, Kaliskiego 2, 01-489 Warsaw, Poland; tomasz.majewski@wat.edu.pl (T.M.); ryszard.wozniak@wat.edu.pl (R.W.); judyta.sienkiewicz@wat.edu.pl (J.S.); 3 Faculty of Chemistry, University of Bialystok, Ciolkowskiego 1K, 15-245 Bialystok, Poland

**Keywords:** sintered iron, molybdenum disulfide, tungsten disulfide, hexagonal boron nitride, lubricant additives, friction

## Abstract

The purpose of the conducted experiments was to test the selected properties of materials intended for porous sintered bearings containing layered materials in the form of powders with an average particle size of 0.5–1.5 μm, with very good tribological properties. The subject of the research was a sinter based on iron powder with the addition of layered materials; molybdenum disulfide MoS_2_ (average particle size 1.5 μm), tungsten disulfide WS_2_ (average particle size 0.6 μm), hexagonal boron nitride, h-BN (average particle size 0.5 and 1.5 μm) with two different porosities. The article presents the results of density and porosity tests, compressive strength, metallographic and tribological tests and the assessment of changes in the surface condition occurring during the long storage period. The use of layered additives allows for an approximately 20% lower coefficient of friction. In the case of sulfides, the technological process of pressing 250 MPa, 350 MPa, and sintering at a temperature of 1120 °C allows us to obtain a material with good strength and tribological properties, better than in the case of h-BN. However, the main problem is the appearance of elements from the decomposition of sulfide compounds in the material matrix, which results in rapid material degradation. In hexagonal boron nitride, such disintegration under these conditions does not occur; the material as observed does not degrade. In this case, the material is characterized by lower hardness, resulting from a different behavior of hexagonal boron nitride in the pressing and sintering process; in this case, pressing at a pressure of 350 MPa seems to be too low. However, taking into account that even with these technological parameters, the obtained material containing 2.5% h-BN with an average grain size of 1.5 μm allowed obtaining a coefficient of friction at the level of 0.41, which, with very good material durability, seems to be very positive news before further tests.

## 1. Introduction

When looking for new solutions to improve friction elements’ cooperation, attention is not always paid to the type of lubricant additives that guarantee a very low coefficient of friction. There is a whole group of additives improving lubricity, introduced to the lubricating oil, or the material of which the elements directly cooperating in the friction system are made. A group of such additives is called layered materials, which include, among others, molybdenum disulfide (MoS_2_) [1], tungsten disulfide (WS_2_) [2], hexagonal boron nitride (h-BN), or graphite (C), et cetera [3].

Tungsten disulfide WS_2_ is an inorganic chemical compound from the sulfides group containing 74.14% tungsten and 25.86% sulfur. Tungsten disulfide belongs to the group of materials characterized by the lowest dry friction coefficient of 0.03. It is a soft material with a layered crystal structure and high thermal stability [4].

Molybdenum disulfide MoS_2_ is insoluble in water; however, it can absorb moisture, which fosters oxidation to MoO_3_. It is a material that achieves a very low coefficient of friction, up to 0.003 [1]; however, this feature deteriorates significantly during its oxidation. It works well, especially in a vacuum.

Hexagonal boron nitride is an inorganic chemical compound obtained by synthesis. It was first synthesized in 1842 by William H. Balmain [4]. Currently, it is received in high-energy methods of producing boron-nitrogen bonds. It comes in three crystallographic varieties (a, b, g). Type a has a hexagonal structure similar to graphite; the name h-BN-hexagonal boron nitride is used to define it. It is a soft, lamellar variety of boron nitride with high anisotropy, used as a lubricant. It is chemically, very stable. Its coefficient of friction is in the range of 0.15–0.7.

The advantage of layered materials is that the atoms of individual elements are bound together by strong covalent bonds within one layer. In contrast, the separate layers are bound by weak Van der Waals forces. This mechanism allows the layers to slide easily over each other, thus ensuring very good tribological properties [4]. These properties result from the formation of plates of layered material on the tribological node’s cooperating friction surfaces. This favors the formation of a low coefficient of friction, confirmed by many tests (footnotes) [5].

Each of these materials has specific properties that are often unfavorable in some applications. The low coefficient of friction, the lack of negative environmental impacts, relatively low cost, or complete application may be more important. Previous studies [6] show that hexagonal boron nitride (h-BN) can be a good lubricant additive. However, it has a slightly higher friction coefficient than molybdenum disulfide or tungsten disulfide, which is cheaper and can be used at much higher temperatures. Simultaneously, it is harmless to health (used, among others, in cosmetics) and non-staining, layered material [7,8,9]. There are several possibilities for introducing a layer agent into the friction node. It can be introduced into the material from which the friction elements are made in the process of its production, for example, by sintered powder metallurgy technology, as coating of friction surfaces (e.g., laser coating). Whole surfaces or only selected parts of them can be coated [10]. Another method of delivering a layered lubricant to the lubrication node is its introduction as a lubricating oil [11,12]. In all cases, the aim is to ensure that the layered lubricant is on the friction surfaces during the tribological node’s operation. The introduction of such additives may reduce friction and have a negative impact on the operating processes. Deterioration of tribological properties in the case of sulfides may be caused by the adsorption of water on the surface and its diffusion into the lubricating film [13]. This problem does not concern, for example, hexagonal boron nitride. The unit price of such an additive cannot be omitted either, as it may impact the cost of the final product. Thus, it is important to select the type, assortment, location, and amount of additive.

The materials used in the tests are widely used, not only as slide bearings, but above all as special bearings, intended for operation at high temperatures without oil impact, or in cases (in medicine) where we want to avoid contamination; an interesting application is, for example, material for the acetabulum of the hip joint [4,8,9]. There are many prospective applications, but before specifying them, it is necessary to investigate such materials and refine their production technologies so that their behavior can be thoroughly understood.

The conducted research tries to answer some of these questions. For this purpose, two stages of research were carried out. One of them concerns the layered lubricant additive’s influence on the selected properties placed in the iron measuring sample during its production using the sintered powder technology. In this case, layered materials with an average particle size of approximately 1 μm were used. In the other part of the research, a layered lubricant’s influence as an additive to lubricating oil was assessed. In this case, the layered materials with a particle size of several dozen nm were used.

This article’s research results in experiments aimed at analyzing the microstructure and decomposition of lubricating additives in the obtained materials and determining their selected mechanical and tribological properties.

## 2. Materials and Methods

The work contains the results of tests of samples made by sintering of iron powder with layered additives improving lubricity (WS_2_, MoS_2_, h-BN) pressed at two pressures (250 MPa, 350 MPa).

As part of the research, density and porosity tests, metallographic tests, strength tests, and selected tribological tests were performed.

### 2.1. Test Equipment

The following equipment was used to prepare the samples: a Fritsch-Pulverisette 5 ball mill, an LPR-25 laboratory hydraulic manual press by Testchem with a die and a punch, allowing obtaining a working pressure of the piston up to 25 kN, a tube furnace model RO 13.5 by VEB Elektro-Industrieofenbau Römhild allowing obtaining a temperature of 1350 °C with a connection power of 13.5 kW and the use of a protective atmosphere in the sintering process.

Compressive strength tests were carried out on the MTS Criterion 45 testing machine equipped with the MTS Test Suite software. This machine, equipped with an electromechanical drive, provides strength tests in the load range from 1 to 100 kN at the traverse speed from 0.005 to 750 mm/min. MTS Test Suite software facilitates the accurate and repeatable mechanical testing of materials, analyzing data, and reporting results

Tribological tests were carried out on a test set to evaluate the UNMT surface layer, which allows for a comprehensive assessment of the mechanical and tribological properties of thin layers and solid materials. Examination of the surface condition using a Nikon ECLIPSE LV100 optical microscope equipped with a NIS-AR computer image analyzer.

A Tegramin-25 grinder-polisher was used to grind and polish the mounted samples, offering solutions allowing for optimal preparation of samples for metallographic tests.

The microscopic examinations were carried out on a scanning electron microscope by Thermo Fisher Scientific—Phenom ProX. This microscope is an advanced model that features an integrated nitrogen-free EDS detector of the SDD type. This microscope is equipped with an extremely efficient CeB6 cathode.

### 2.2. Test Materials

The samples were prepared from iron powder with the addition of a layered lubricant. They were produced according to the following manufacturing process: preparation of powder mixtures, matrix pressing (pressing pressure 250 or 350 MPa) and they were held at the highest pressure for 1 min, sintering in a tube furnace (protective atmosphere: dissociated NH3, sintering temperature: 1120 °C, sintering time: 1 h), and machining. The condition of the sintering process was set based on the available literature [6,14].

#### 2.2.1. Iron Powder

SC 100.40 iron powder by Högänas Sweden AB was used to make the samples. Its basic properties are shown in Table 1. It is one of the most widely used powders for the production of sintered parts. An irregular shape, a porous structure of particles characterizes it, and it has a low oxygen content. It is easy to press, and the obtained parts have high compressive strength. (http://haganas.com/).

#### 2.2.2. Layered Lubricating Additives

During the preparation of the test samples, layered lubricating additives were added to the iron powder: tungsten disulfide WS_2_ (0.6 μm), molybdenum disulfide MoS_2_ (1.5 μm), and hexagonal boron nitride h-BN (0.5 μm, 1.5 μm). The basic properties of layered lubricating materials used in investigations were presented in Table 2.

### 2.3. Preparation of Samples for Research

In the first stage of the planned tests, samples were prepared according to the following variants: two different pressing pressures were used, selected to obtain porosity of about 17% and about 26%. The powder mixes were made on a Fe powder with the addition of hexagonal boron nitride (h-BN) with an average grain size of 0.5 μm and 1.5 μm, or molybdenum disulfide (MOS_2_) with an average grain size of 1.5 μm, or tungsten disulfide (WS_2_) with an average grain size of 0.6 μm at concentrations of 0.5%, 2.5%, and 5% by weight, respectively. For this purpose, powder mixtures were prepared, and the pressing and sintering process was carried out. A 1% zinc stearate lubricant (trade name Kenolube) was incorporated into the blend containing only Fe powder for better compressibility. Before pressing, tests were carried out to determine the pressing pressure so as to obtain samples with the expected porosity after sintering.

#### 2.3.1. Making a Powder Mixture

Powder mixtures were made according to the adopted variants. They were poured into mortars, and then 80 g of alcohol was added to each. The next step was to mix the powders in a ball mill for 1 h to obtain a homogeneous blend. Mortars were then placed in an oven, where the alcohol was evaporated.

#### 2.3.2. Making Samples

In the next stage, green compacts were made using the method of matrix pressing. For this purpose, a matrix was used with a punch (presser), into which a weighed portion of individual powder mixtures was poured. With the use of a hydraulic press, the compacts were prepared using a pressing pressure of 250 MPa for samples with the assumed porosity of 26% and 350 MPa for samples with the assumed porosity of 17%; these pressures were selected based on successive experiments for individual powder mixtures. To standardize the production conditions, the reference base was the set pressing pressure.

After determining their density and porosity, the green compacts were subjected to a sintering process. A tube furnace was used for this process, in which materials were made in the presence of a protective atmosphere of dissociated ammonia.

### 2.4. Research Methodology

#### 2.4.1. Compressive Strength Tests

Compressive strength tests were carried out using the MTS Criterion 45 universal testing machine with the MTS Test Suite software. The samples were subjected to longitudinal compression by ASTM E9-09 standard. Samples had a cylindrical shape and dimensions of f6 × 9 mm. There were three samples of each kind of material tested as condition of the manufacturing process. The strain was measured by extensometry, and the strain rate 5 × 10^−3^ (m/m·min) was used in the tests.

Thanks to the coupling of the testing machine with a PC, it was possible to register both the pressure force and the samples’ shortening. The results were saved in the form of a text file, and based on them, a diagram showing the course of the compression process was developed.

Determination of the tested samples’ yield strength (R0,2) was made automatically by applying the procedure included in the MTS software (version no. 2.3.1).

#### 2.4.2. Metallographic Research

Samples for metallographic tests were ground on a STRUERS TEGRAMIN grinding-polisher using 80–4000 abrasive papers and then polished mechanically using a diamond suspension of 9 and 3 µm and a silica suspension with a grain size of 0.25 µm. The microscopic observations were performed on a Phenom ProX scanning electron microscope. The qualitative and quantitative analysis of the selected micro-areas of the obtained samples was performed using the EDS (energy dispersive X-ray spectrometry) analyzer.

#### 2.4.3. Tribological Tests

Friction tests were carried out for pairs of samples in a sphere-to-plane combination, in reciprocating motion, without lubrication, based on the conditions specified in the ASTM G-133 standard.

The experiment parameters were as follows:

- ball material: tungsten carbide (WC);
- ball diameter: 3.175 mm (1/8”);
- load: 2.79 N (deviation from ASTM G-133 due to the size of the ball, providing pressures as in ASTM G-133);
- amplitude: 2 mm (deviation from ASTM G-133 due to sample size);
- test duration: 1000 s (by ASTM G-133);
- frequency: 5 Hz (by ASTM G-133);
- the total number of cycles: 5000 (by ASTM G-133); and
- friction length: 20 m (deviation from ASTM G-133 due to reduced amplitude).

Friction tests were used to determine the friction coefficient (based on the recorded friction force) between the sample and the counter-sample (ball with WC).

#### 2.4.4. Assessment of the Surface Appearance of Samples

The samples’ surface condition was assessed by comparing the appearance (surface condition) with other samples that had been stored for over a year under the laboratory’s atmospheric conditions. This condition was recorded with a camera.

## 3. Results and Discussion

As part of the numerous studies carried out, numerous interesting results were obtained, but their number does not include all of them. Only those that are of decisive importance for evaluating the research and confirm the assumptions made will be mentioned.

### 3.1. The Results of the Density and Porosity Tests

**Figure 1:**
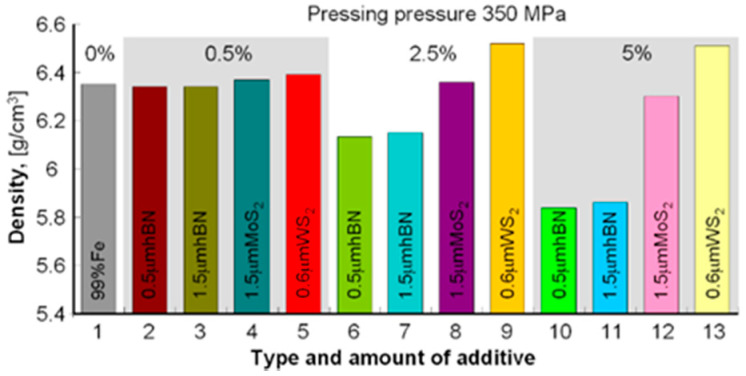
Sample densities after pressing 350 MPa and sintering.

**Figure 2:**
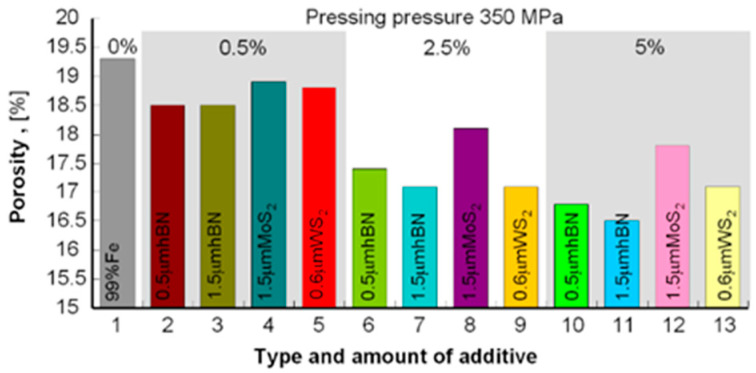
Sample porosity after pressing 350 MPa and sintering.

**Figure 3:**
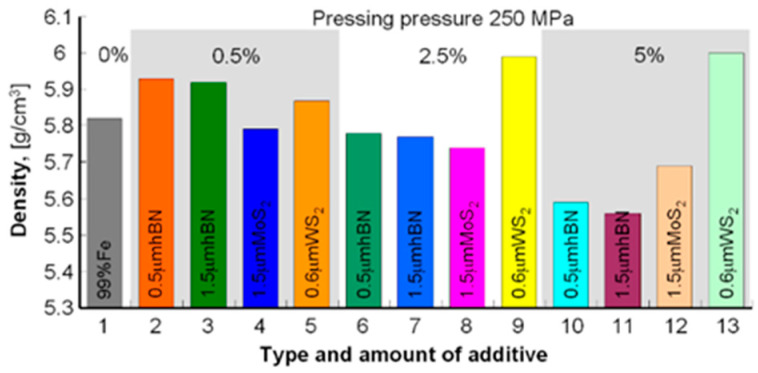
Sample densities after pressing 250 MPa and sintering.

**Figure 4:**
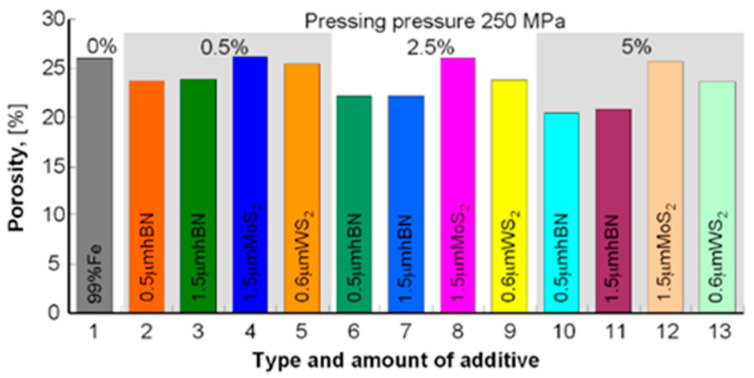
Sample porosity after pressing 250 MPa and sintering.

The samples’ density was measured by the Archimedes method (mass measurement in air and water). To avoid wetting the samples before measurement in water, they were protected with a thin layer of petroleum jelly. Samples with a density of 5.56–6.00 g/cm^3^ were obtained for a pressure of 250 MPa and 5.84–6.52 g/cm^3^ for the pressure of 350 MPa; the results are summarized in Table 3 and to compare the obtained densities and porosities in the diagrams in Figure 1, Figure 2, Figure 3 and Figure 4.

The presented results show that using the h-BN additive, both for the 0.5 μm and 1.5 μm powder, similar density results were obtained while increasing the amount of this additive in the sinter resulted in lower porosity in both cases. Similar dependencies can be seen in using MoS_2_ and WS_2_ additives, although their effect on porosity is smaller than that of h-BN. In general, it can be concluded that the highest porosity was obtained for Fe + Kenolube materials and the lowest for Fe + h-BN materials. It can also be concluded that layer additives play the role of slip additives in the pressing process.

### 3.2. Strength Test Results

**Figure 5:**
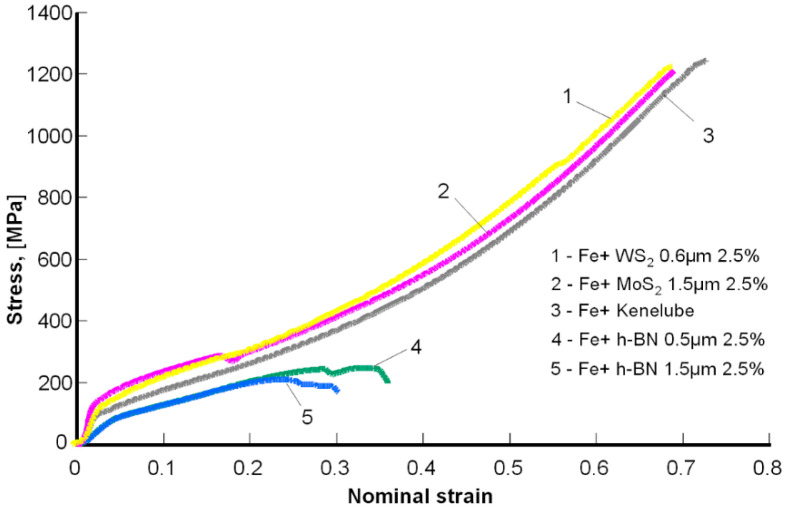
Compression diagram for a Fe sample with layered additives.

**Figure 6:**
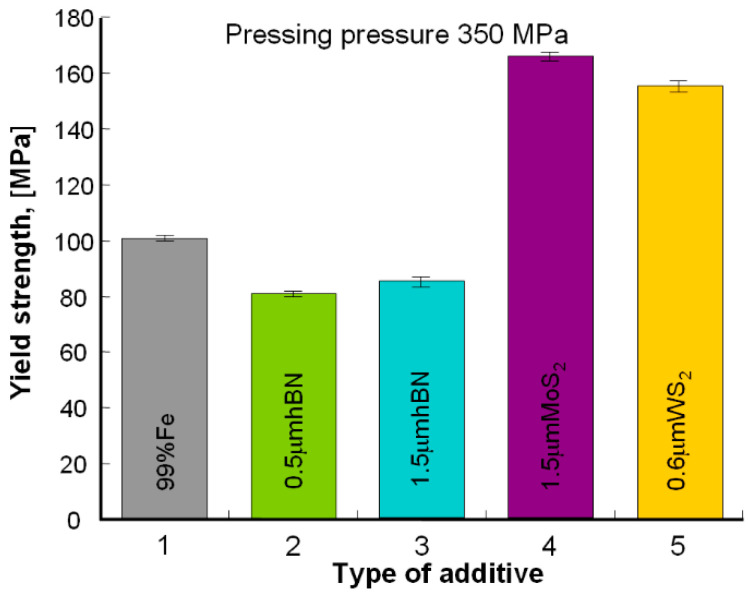
Results of compression measurements of sintered samples pressed under the pressure of 350 MPa.

**Figure 7:**
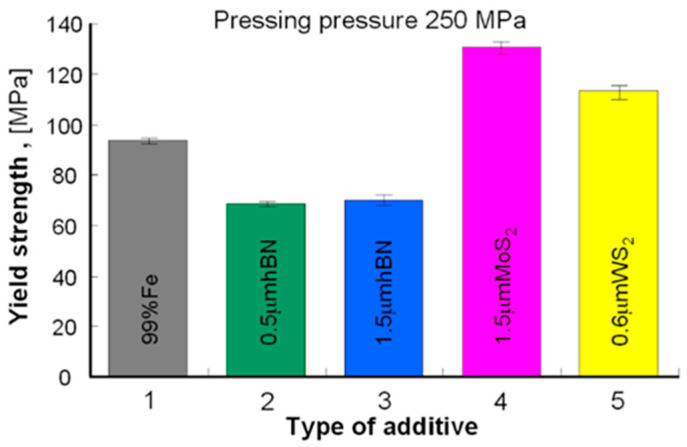
Results of compression measurements of sintered samples pressed under the pressure of 250 MPa.

Figure 5 presents examples of the tested samples results in the form of nominal stress vs. nominal strain, and Table 4—the results of the yield strength measurements, the comparison of which is presented in Figure 6 and Figure 7.

As can be seen from the presented results (Figure 6 and Figure 7), the highest value of the yield strength was found in the Fe sintered with WS_2_ and MoS_2_, and the lowest—Fe sintered with the addition of h-BN. In each case, the materials pressed under higher pressure (with lower porosity) showed a higher yield strength value.

In contrast, the lowest plasticity (determined by the shortening value) was observed in Fe sintered with the addition of boron nitride (approximately 0.3). The remaining samples did not show any distinct cracks during the compression test and were characterized by much greater plasticity (above 0.7). Therefore, for these materials, it was not possible to determine the compressive strength limit (it was more than 1200 MPa). However, it can be concluded that the R_c_ value for Fe sintered with the addition of boron nitride did not exceed 250 MPa.

### 3.3. Metallographic Test Results

**Figure 8:**
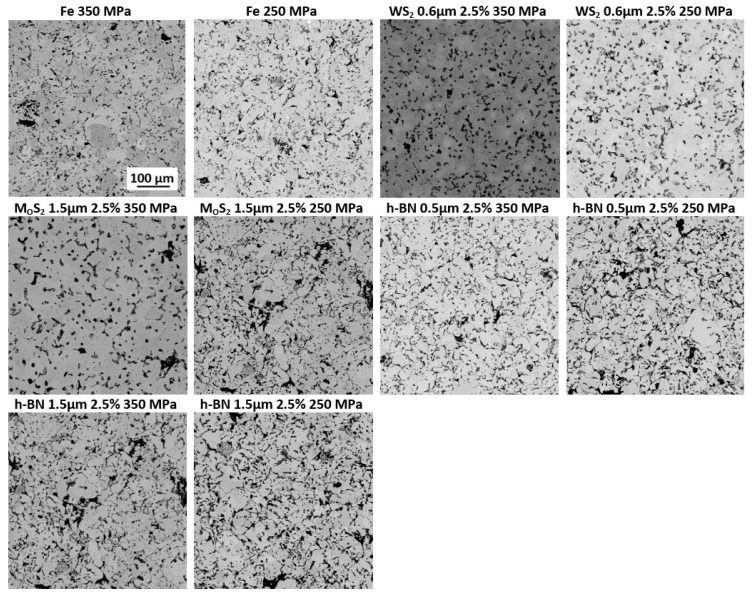
Scanning electron micrograph showing microstructures of obtained samples (magnification 500×).

Figure 8 shows images of the obtained samples’ microstructures, pressed before sintering at the pressures of 350 MPa and 250 MPa. The photos were taken at 500 × magnification. The porosity in the SEM photos is visible as clear black spaces between the sintered powder particles. It can be seen that the pore size is generally small (below 10 µm). Nevertheless, places where the pore size is up to 50 µm, are visible. The pores found in all samples are irregular in shape. In the group of samples pressed at a pressure of 350 MPa, the porosity differences are small and distributed similarly in each of the materials. However, samples containing h-BN have more partially connected pores. Such a phenomenon is more evident for samples pressed under a pressure of 250 MPa, which are more porous.

The performed qualitative and quantitative EDS analysis of selected micro-areas of all samples is shown in Figure 9, Figure 10, Figure 11, Figure 12, Figure 13, Figure 14, Figure 15 and Figure 16.

**Figure 9:**
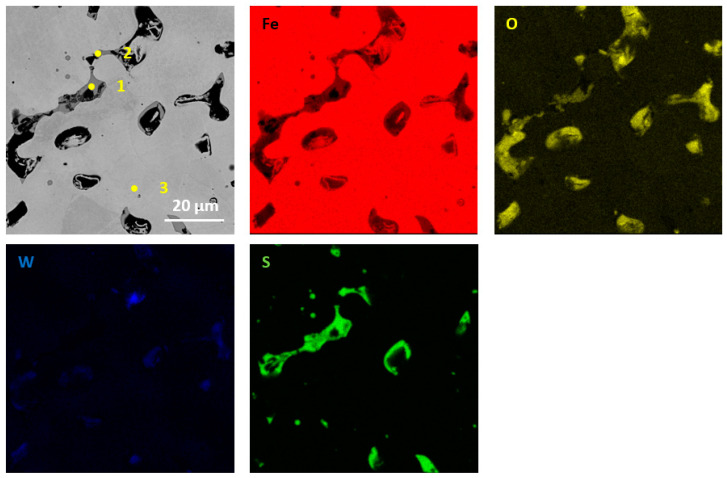
EDS mapping of sintered Fe with the addition of WS_2_ with a size of 0.6 µm (for pressing pressure 350 MPa) together with point analysis.

**Figure 10:**
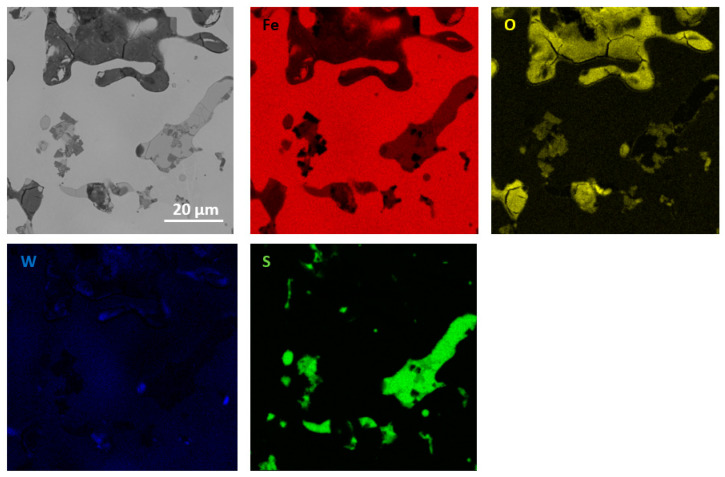
EDS mapping of sintered Fe with the addition of WS_2_ with a size of 0.6 µm (for pressing pressure 250 MPa).

The microstructure of iron sintered with the addition of tungsten disulfide WS_2_ is shown in Figure 9 and Figure 10. The pores occur mainly at the boundaries of the sintered particles. Additionally, tungsten disulfide particles are visible. The chemical composition at selected points (points 1–3 in Figure 9) is presented in Table 5. As can be seen, both sulfur and tungsten are present in the matrix material only in a negligible amount (point 3). In contrast, while increased sulfur content can be noticed at the matrix grain boundaries. This may prove that there was no diffusion of sulfur and tungsten into the iron matrix [15,16].

**Figure 11:**
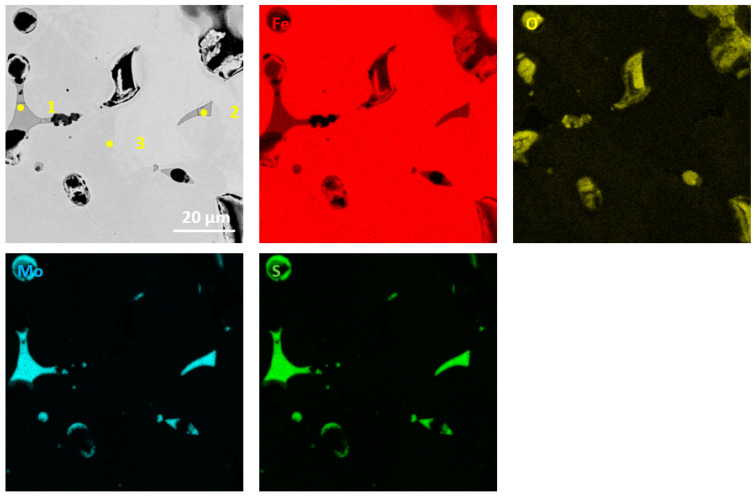
EDS mapping of sintered Fe with the addition of MoS_2_ with a size of 1.5 µm (for pressing pressure 350 MPa).

**Figure 12:**
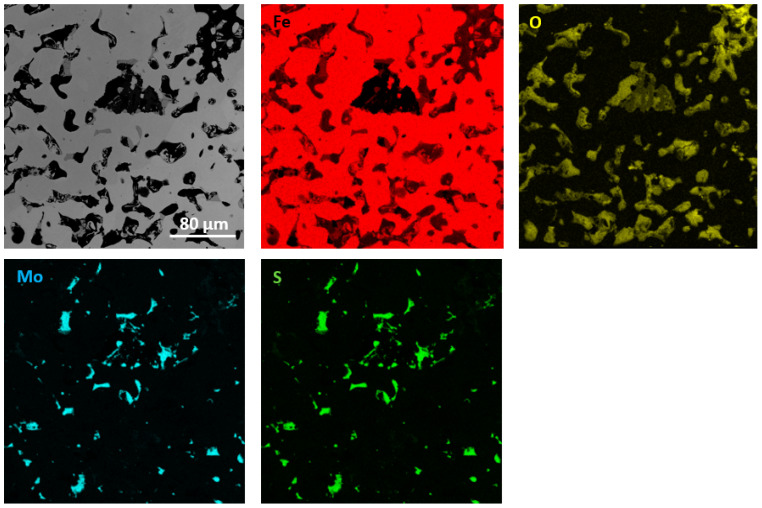
EDS mapping of sintered Fe with the addition of MoS_2_ with a size of 1.5 µm (for pressing pressure 250 MPa).

In Figure 11 and Figure 12, elements mapping for Fe sintered with the addition of MoS_2_ with a size of 1.5 µm (for two pressing pressures) has been shown. The higher magnification image (Figure 11 and Figure 12) exhibits a two-phase structure for both materials composed of a Fe matrix and MoS_2_ particles. Chemical analysis showed that Fe is present in the MoS_2_ particles. The materials are likely to have a ferritic microstructure with the second phase homogeneously dispersed in the matrix. Based on the available literature, it is known that molybdenum disulfide decomposes in sintered iron, and molybdenum additionally stabilizes the ferritic structure [17,18,19,20]. The chemical composition at selected points (points 1–3 in Figure 11) is presented in Table 6.

**Figure 13:**
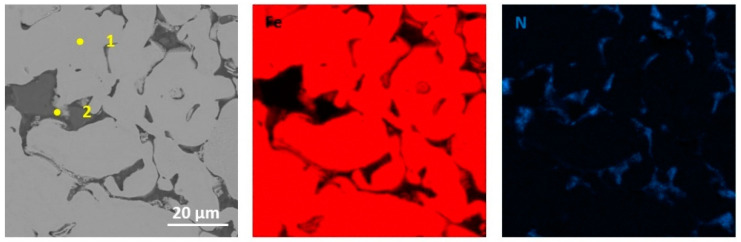
EDS mapping of sintered Fe with the addition of h-BN with a size of 0.5 µm (for pressing pressure 350 MPa).

**Figure 14:**
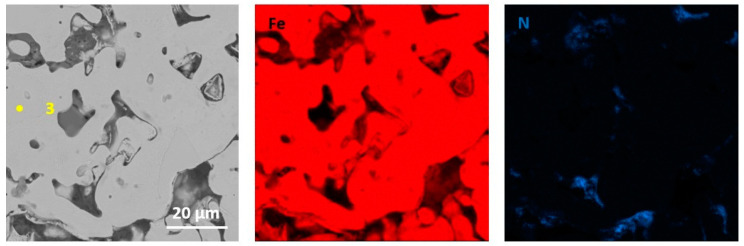
Figure 7 EDS mapping of sintered Fe with the addition of h-BN with a size of 0.5 µm (for pressing pressure 250 MPa).

**Figure 15:**
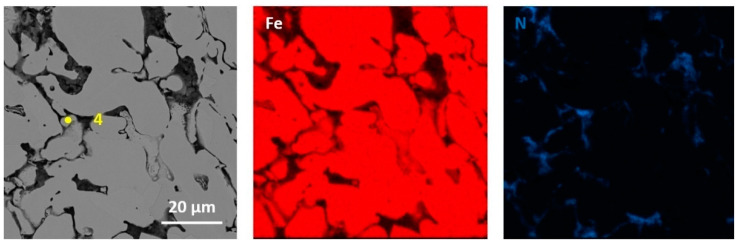
EDS mapping of sintered Fe with the addition of h-BN with a size of 1.5 µm (for pressing pressure 350 MPa).

**Figure 16:**
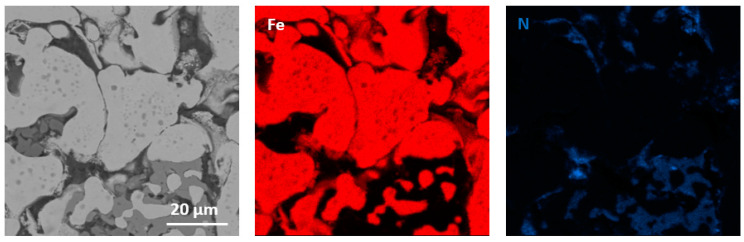
EDS mapping of sintered Fe with the addition of h-BN with a size of 1.5 µm (for pressing pressure 250 MPa).

Figure 13, Figure 14, Figure 15 and Figure 16 present elements mappings, respectively, for sintered Fe with 2.5% h-BN with a particle size of 0.5 µm (pressed at 350 MPa pressure), Fe with 2.5% h-BN with a particle size of 0.5 μm (pressed at 250 MPa pressure), sintered Fe with 2.5% h-BN with a particle size of 1.5 µm (pressed at 350 MPa pressure), and sintered Fe with 2.5% h-BN with particle size of 1.5 µm (pressed at 250 MPa pressure). The particles of hexagonal boron nitride h-BN are present at the grain boundaries. It was observed that neither boron nor nitrogen is present in the Fe matrix. This suggests that the h-BN phase is stable [21,22]. The chemical composition at selected points (points 1–4 in Figure 13, Figure 14 and Figure 15) is presented in Table 7.

Based on the microstructural analysis, it was found that only for hexagonal boron nitride h-BN, regardless of whether it was the pressing pressure higher—350 MPa or lower—250 MPa, was there no collapsing of boron nitride. In the case of tungsten disulfide, WS_2_, and molybdenum disulfide MoS_2_, the presence of tungsten, molybdenum, and sulfur in the matrix material indicates the decomposition of WS_2_ and MoS_2_.

### 3.4. Results of Tribological Tests

The tribological tests aimed to determine the value of the friction coefficient for materials formed in various configurations. They were carried out according to the procedure described in Section 2.4.3. The results of these experiments are presented in Table 8.

**Figure 17:**
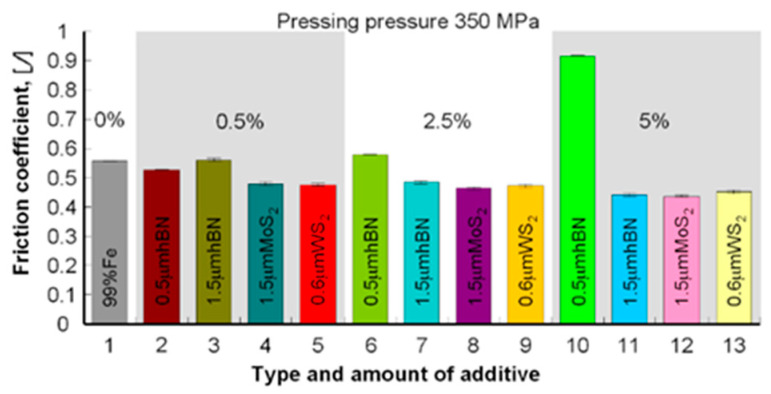
The coefficient of friction depending on the amount and type of layered additive in the samples pressed under the pressure of 350 MPa.

**Figure 18:**
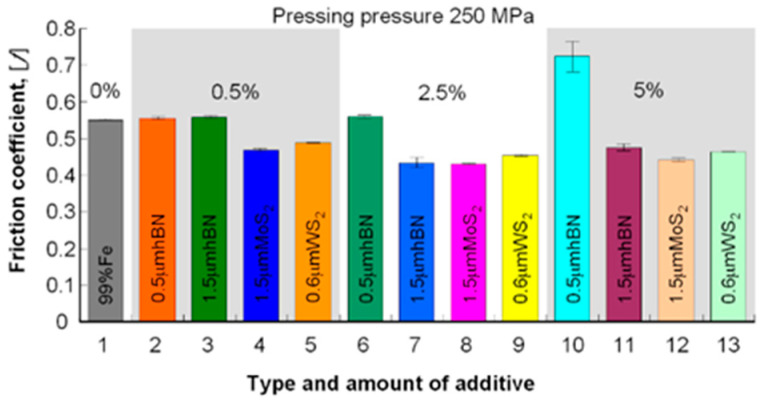
The coefficient of friction depending on the amount and type of layered additive in the samples pressed under the pressure of 250 MPa.

During the comparative analysis of samples with layered lubricating additives (WS_2_, MoS_2_, h-BN), an exact positive effect of these additives on the friction coefficient’s value can be seen in comparison with the starting material (Fe). Only in one case, the tested materials’ friction coefficient is higher than the Fe friction coefficient (0.5 μm h-BN). In other cases, it is lower or similar (Table 8, Figure 17 and Figure 18).

**Figure 19:**
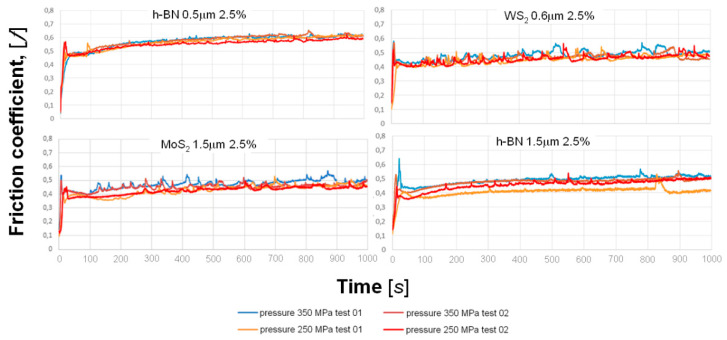
Function sequence in the friction coefficient during the test.

During the entire test (1000 s), apart from the so-called start-up, slight changes in the friction coefficient can be observed. It can be seen in the example graphs shown in Figure 19.

There was also a slight decrease in the resistance to motion, along with the increasing concentration of tungsten disulfide WS_2_ (from about 0.48 to about 0.45). Simultaneously, the lowest value was obtained for samples pressed at a pressure of 250 MPa and 2.5% of the lubricant additive (0.45), and the highest value for samples pressed at a pressure of 250 MPa and 0.5% of the additive. 

The friction coefficient shows similar dependencies for all samples with the addition of MoS_2_ molybdenum disulfide—irrespective of the concentration of the additive and the porosity of the sinter. The lowest value of the friction coefficient was obtained for samples pressed at a pressure of 250 MPa and 2.5% of lubricant additive (0.43), and the highest value for samples pressed at a pressure of 350 MPa and 0.5% of the additive.

**Figure 20:**
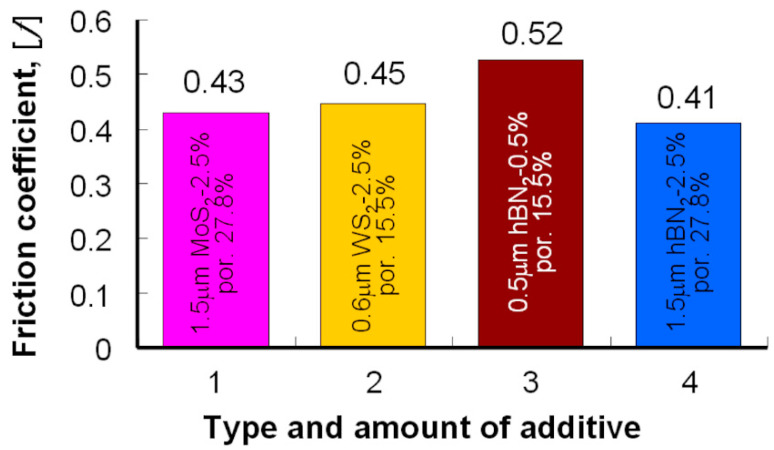
The lowest value of the friction coefficient obtained for individual additives.

The changes of the friction coefficient for all samples with the addition of h-BN were quite similar—the samples with the addition of 2.5% h-BN showed the highest stability of resistance to motion, for the grain size 0.5 μm. An interesting case that requires further analysis is the use of the h-BN additive, especially with a grain size of 0.5 μm. The observation shows that the worst results obtained in this case may be due to the problems that appear during the pressing and sintering of iron powder with the addition of h-BN. The lowest values of the friction coefficient achieved during the tests for all used layer additives are shown in Figure 20.

### 3.5. Assessment of the Appearance of Samples after Their Storage

**Figure 21:**
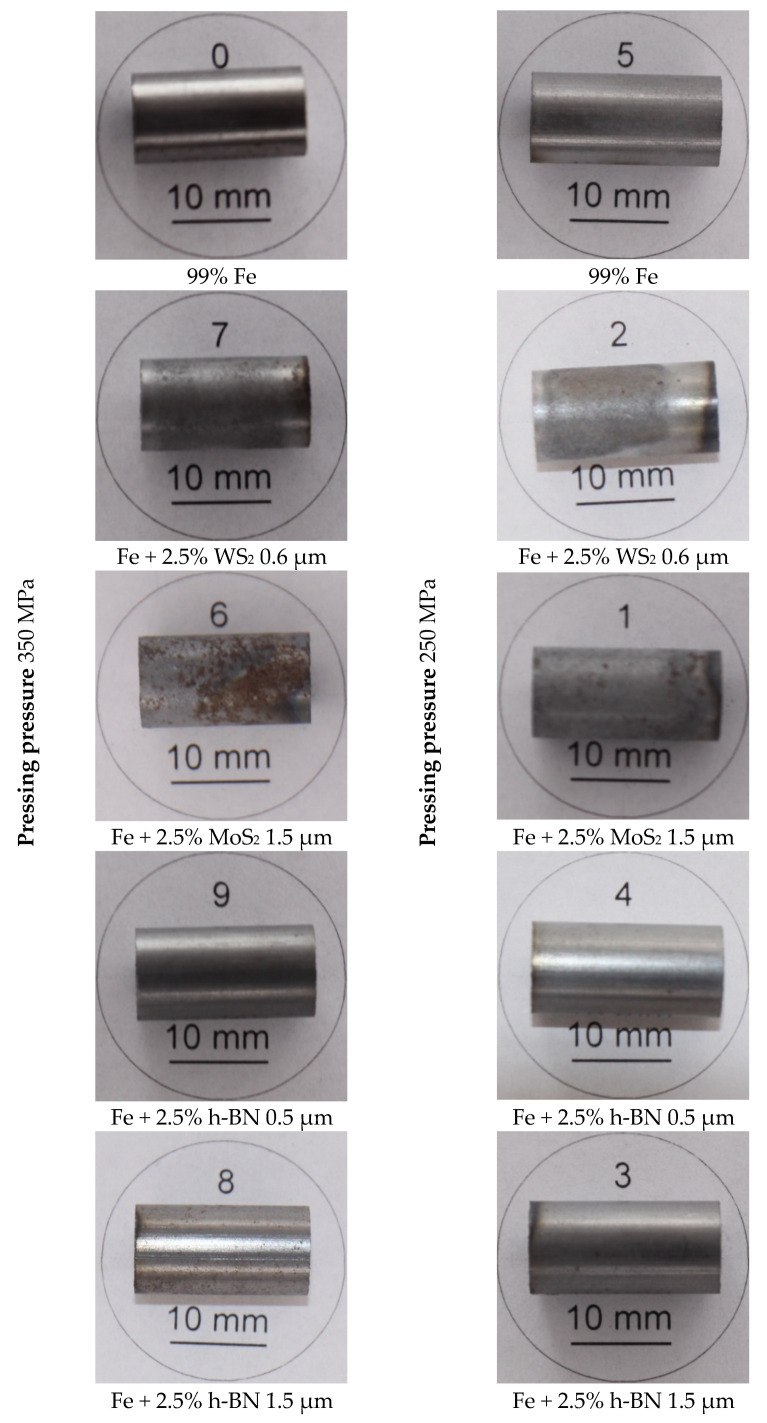
The appearance of the samples after keeping them in the room for one year.

Below are photos of selected samples that show the samples’ appearance after they have been stored for about one year in a laboratory room under atmospheric conditions: temperature 18–22 °C and humidity 40–60 RH. As observed, the samples’ surfaces containing the addition of MoS_2_ and WS_2_ became oxidized, which significantly increased their roughness (Figure 21). The more of this additive the samples had, the worse the surface quality was and the more powder was released from it around the samples. In the samples with h-BN, such changes were not noticed; the samples did not differ in appearance from the samples made without a lubricant. This is probably due to the sulfur’s action emitted from the sulfides, which degrades the surface. It has been described in publications [23,24]. The assessment of the surface condition may be the direction of further research.

## 4. Conclusions

The work’s main purpose was to check how the type, quantity, and grain size of selected layered materials (MoS_2_, WS_2,_ h-BN) affects the functional properties (density and porosity, friction, compressive strength, susceptibility to atmospheric air) of sintering.

The type of additive used, its amount, and grain size significantly impact the obtained material from the very beginning of its production. In the cases of pressing at a pressure of 250 MPa or 350 MPa, the introduction of a MoS_2_ and WS_2_ additive has a positive effect on the mechanical properties (compressive strength) with samples made without lubricant additives, which may be related to the slightly higher porosity of the latter. When using the h-BN lubricant additive, the compressive strength and yield strengths of such samples were much lower than in other samples.

Tribological tests have shown that the introduction of layered lubricant additives reduces the friction coefficient; they also confirm that the grain size has a smaller effect on this change. The amount of this additive has a significant impact; the optimal composition is 2.5%. Comparing the results obtained for individual additives, it can be concluded that the best of them was obtained for MoS_2_ and WS_2_; excellent results were also obtained for 1.5 μm h-BN, which is a very interesting result with much lower yield strength for these samples. The introduction of these additives with the appropriate technology of sample production allows us to achieve a 20% lower coefficient of friction.

At the final stage of the research, all samples were left under the influence of the atmospheric air in the room, and the change in the appearance of their external surface was observed. The observations show that the most traces of oxidation were shown by samples made with the addition of MOS_2_ and WS_2_; the surface condition was even worse than that of samples made only with iron powder. The samples containing h-BN behaved similarly, without noticeable changes on the surface.

## 5. Concluding Remarks

Taking into account the obtained results, it can be concluded that the introduction of layered lubricant additives to the material from which the samples for sintered bearings are made may be a controversial solution. Although they allow obtaining about 20% lower friction coefficient, economic and operational considerations limit such use. Looking for the optimal solution, 1.5 μm h-BN can be used as a lubricant additive in the amount of 2.5%, however, on condition that the technology of producing such materials is refined, so that the manufacturing process allows obtaining the best properties. The advantage of such a solution may be that h-BN, which is several times cheaper than other additives, gives the possibility of obtaining a very low friction coefficient (even more than 20% lower than in the case of a pure iron sample, while being chemically inert it does not have a destructive effect on the material from which the steel bearing is made, which cannot be said about sulfide additives.

## Figures and Tables

**Table 1:** SC 100.40 iron powder.

Approximate Particle Size Range µm	Apparent Density g/cm^3^	Flow s/50g	H_2_-loss %	C %	Green Density g/cm^3^
45–150	2.45	32	0.14	< 0.01	7.1

**Table 2:** Layered materials.

Properties	MoS_2_	WS_2_	h-BN
Molecular weight (g/mol)	160.9	248	24.82
Metallic properties	non-metal	non-metal	non-metal
Appearance	dark	gray	white
Mohs scale of hardness	1 ± 1.5	0.5 ± 0.75	1.5 ± 2
Density, (g/cm^3^)	5.06	7.5	1.7 ± 2.2
Melting temperature, (°C)	1185	1250	2973
Lubrication temperature range, (°C)	185 ± 350	from −273 to 650	−40 ± 870
Coefficient of friction (min)	<0.1	0.07	0.15–0.7

**Table 3:** Results of density and porosity tests.

Type of Sinter	Additive Content (% mass)	Pressing Pressure (MPa)			
		350		250	
		Density (g/cm^3^)	Porosity (%)	Density (g/cm^3^)	Porosity (%)
Fe + Kenolube	0	6.35	19.3	5.82	26.1
Fe + h-BN 0.5 μm	0.5	6.34	18.5	5.93	23.7
Fe + h-BN 1.5 μm	0.5	6.34	18.5	5.92	23.9
Fe + MoS_2_ 1.5 μm	0.5	6.37	18.9	5.79	26.2
Fe + WS_2_ 0.6 μm	0.5	6.39	18.8	5.87	25.4
Fe + h-BN 0.5 μm	2.5	6.13	17.4	5.78	22.2
Fe + h-BN 1.5 μm	2.5	6.15	17.1	5.77	22.2
Fe + MoS_2_ 1.5 μm	2.5	6.36	18.1	5.74	26.1
Fe + WS_2_ 0.6 μm	2.5	6.52	17.1	5.99	23.8
Fe + h-BN 0.5 μm	5	5.84	16.8	5.59	20.4
Fe + h-BN 1.5 μm	5	5.86	16.5	5.56	20.8
Fe + MoS_2_ 1.5 μm	5	6.30	17.8	5.69	25.7
Fe + WS_2_ 0.6 μm	5	6.51	17.1	6.00	23.6

**Table 4:** A comparison of average measurement results of the yield strength.

Type of Material	Yield Strength (MPa)	
	Pressing Pressure 350 MPa	Pressing Pressure 250 MPa
Fe 99%	102 ± 2%	94 ± 2%
Fe + 2.5% h-BN 0.5 µm	82 ± 2%	69 ± 2%
Fe + 2.5% h-BN 1.5 µm	87 ± 5%	70 ± 5%
Fe + 2.5% M_O_S_2_ 1.5 μm	168 ± 4%	131 ± 3%
Fe + 2.5% WS_2_ 0.6 μm	158 ± 5%	114 ± 5%

**Table 5:** EDS point analysis of a sintered Fe sample (for pressing pressure 350 MPa) containing 2.5% WS_2_ with a grain size of 0.6 µm.

Point	Fe at.% (wt.%)	O at.% (wt.%)	W at.% (wt.%)	S at.% (wt.%)
1	35 (55)	32 (14.22)	-	34 (31)
2	80 (84)	5 (1.34)	2 (7)	14 (8)
3	96 (97)	4 (1.11)	1 (2)	<1 (<1)

**Table 6:** EDS point analysis of a sintered Fe sample (for pressing pressure 350 MPa) containing 2.5% MoS_2_ with a grain size of 1.5 µm.

Point	Fe at.% (wt.%)	O at.% (wt.%)	Mo at.% (wt.%)	S at.% (wt.%)
1	52 (49)	-	23 (37)	26 (14)
2	94 (97)	5 (2)	<1 (<1)	<1 (<1)
3	95 (98)	5 (2)	<1 (<1)	<1 (<1)

**Table 7:** EDS point analysis of sintered Fe with hexagonal boron nitride h-BN.

Point	Fe at. % (wt.%)	O at. % (wt.%)	N at. % (wt.%)
1	85 (96)	4 (1)	0 (0)
2	–	65 (49)	10 (7)
3	72 (92)	6 (2)	–
4	58 (84)	12 (5)	25 (9)

**Table 8:** Results of tribological tests.

Material Composition	Additive Content (% mass)	Pressing Pressure (MPa)/Assumed Porosity (%)	
		250/17	350/26
		The Averaged Value of the Coefficient of Friction	The Averaged Value of the Coefficient of Friction
Fe	0	0.56 ± 0.36%	0.55 ± 0.18%
Fe + h-BN 0.5 μm	0.5	0.53 ± 0.38%	0.56 ± 0.72%
Fe + h-BN 1.5 μm	0.5	0.56 ± 1.43%	0.56 ± 1.00%
Fe + MoS_2_ 1.5 μm	0.5	0.48 ± 1.66%	0.47 ± 0.98%
Fe + WS_2_ 0.6 μm	0.5	0.48 ± 1.89%	0.49 ± 0.21%
Fe + h-BN 0.5 μm	2.5	0.58 ± 0.17%	0.56 ± 1.17%
Fe + h-BN 1.5 μm	2.5	0.48 ± 2.04%	0.42 ± 3.07%
Fe + MoS_2_ 1.5 μm	2.5	0.46 ± 2.27%	0.43 ± 0.40%
Fe + WS_2_ 0.6 μm	2.5	0.47 ± 2.23%	0.45 ± 0.58%
Fe + h-BN 0.5 μm	5	0.91 ± 0.11%	0.72 ± 9.45%
Fe + h-BN 1.5 μm	5	0.44 ± 0.90%	0.47 ± 2.00%
Fe + MoS_2_ 1.5 μm	5	0.43 ± 0.69%	0.44 ± 1.04%
Fe + WS_2_ 0.6 μm	5	0.45 ± 1.32%	0.46 ± 0.22%
